# Enhancing Left Ventricular Assist Device Usability: A Comparative Simulation Study of CorWave and HeartMate 3 Peripherals

**DOI:** 10.1097/MAT.0000000000002472

**Published:** 2025-05-30

**Authors:** Gregor Widhalm, Theodor Abart, Katharina Ebenberger, Angelika Berger, Roxana Moayedifar, Daniel Zimpfer, Julia Riebandt, Michael Wagner, Thomas Schlöglhofer

**Affiliations:** From the * Department of Cardiac and Thoracic Aortic Surgery, Medical University of Vienna, Vienna, Austria; † Division of Neonatology, Pediatric Intensive Care and Neuropediatrics, Department of Pediatrics, Comprehensive Center for Pediatrics, Medical University of Vienna, Vienna, Austria; ‡ Ludwig Boltzmann Institute for Cardiovascular Research, Vienna, Austria; § Center for Medical Physics and Biomedical Engineering, Medical University of Vienna, Vienna, Austria.

**Keywords:** left ventricular assist device, human factors engineering, usability, user-centered design, wearables

## Abstract

Left ventricular assist devices (LVADs) improve outcomes but often compromise quality of life (QoL) due to usability issues with wearables. This study compared the usability of CorWave LVAD (CW) peripherals prototypes to HeartMate 3 LVAD (HM3) peripherals through a cross-sectional, simulation-based approach involving LVAD-naive laypeople, and heart transplantation (HTX) patients post-LVAD support. Simulations encompassed six tasks, measuring initial success rates, duration to success, pump-off time, and a post-scenario survey. Forty-six untrained participants (16 CW *vs.* 30 HM3; 12.5% *vs.* 20% female, *p* = 0.69; 56.2% *vs.* 50.0% HTX patients, *p* = 0.76) completed 276 scenarios. The CW cohort demonstrated superior initial success rates (92.7% *vs.* 80.6%, *p* = 0.008). Battery exchanges (in normal and dim light, within carry bag) were completed twice as quickly for CW users (*p* ≤ 0.001). Although controller exchange success rates were comparable, the duration to success and pump-off times were doubled for the HM3 cohort (*p* ≤ 0.005). During the “connection to AC power” scenario, HM3 subjects experienced higher complexity, as the CW cohort achieved 5× lower duration to success and 3× higher initial success rates (*p* ≤ 0.001). Survey responses favored CW cable lengths (92.9% *vs.* 69.0%, *p* = 0.001). This study highlights the advantages of CW’s user-centered design, which may enhance QoL and safety for future LVAD patients.

## Graphical abstract

**Figure:**
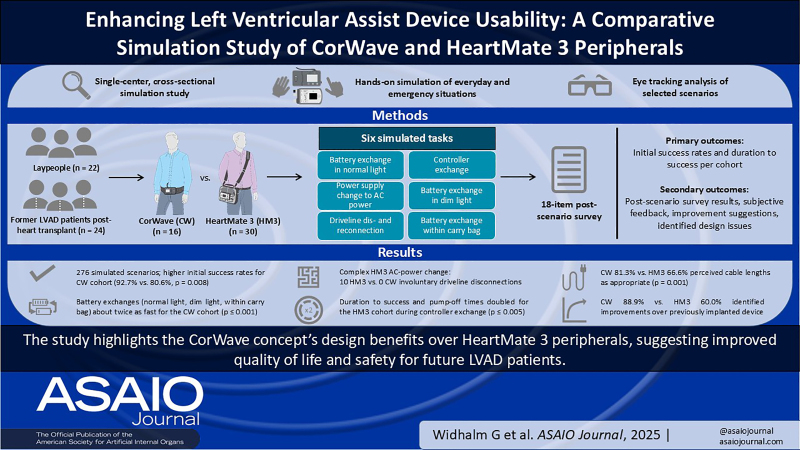

Continuous improvements in left ventricular assist device (LVAD) therapy over recent decades have provided a valuable treatment option for patients with end-stage heart failure, improving both survival and quality of life.^1,2^ The currently clinically available, fully magnetically levitated HeartMate 3 (HM3; Abbott, Chicago, IL), has shown excellent clinical outcomes with 1 and 5 year survival rates of 86% and 64%, respectively.^1^ To address persistent challenges associated with continuous-flow LVADs,^3,4^ including gastrointestinal bleeding events,^5,6^ CorWave SA (CW, Clichy, France) has introduced a new concept of a pulsatile wave membrane pump.^7–10^ Like all previous LVADs, the CW device comes with external peripherals, primarily operated by patients and their caregivers.^11^ Although the daily configuration of the HM3 peripherals requires two batteries discharged in parallel,^12^ the CW concept is based on a single battery clipped on the controller and an additional optional tethered battery. The controller also features a built-in backup battery with a minimal lifetime of 5 min, ensuring continued operation in case of battery exchange. The total battery life, including the tethered battery, is a minimum of 8 h. Furthermore, the CW prototype incorporates a helical driveline, providing additional cable length in case of controller drop. The total weight of the CW peripherals ranges from 1.1 kg in the clip-on battery configuration to less than 1.8 kg when including the tethered battery (see Supplemental Digital Content 1, https://links.lww.com/ASAIO/B522). A comparison of both wearable concepts is provided in Figure 1.

**Figure 1.**
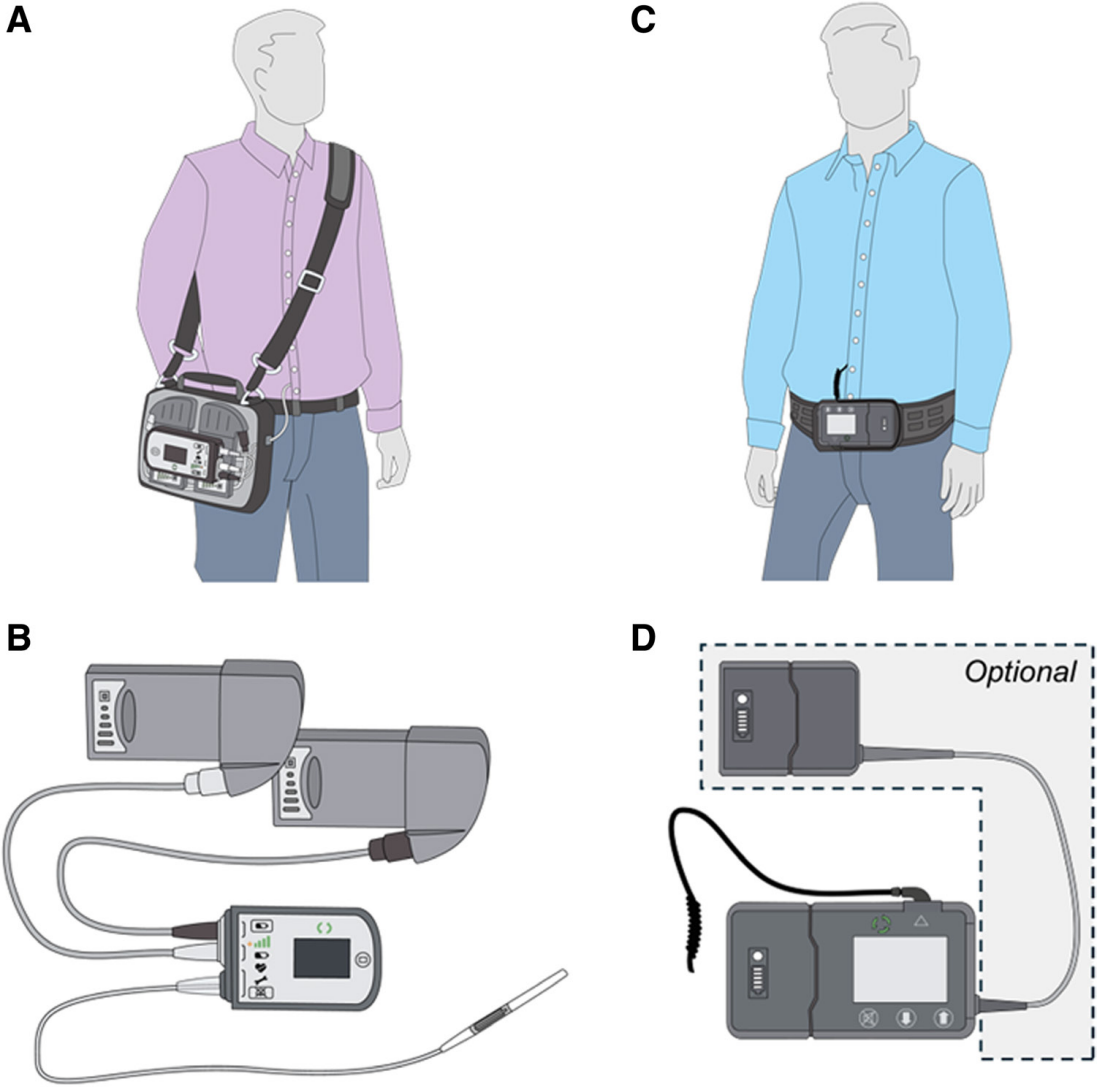
Schematic daily configuration of HeartMate 3 peripherals (**A, B**) and CorWave LVAD peripherals prototype (**C, D**). LVAD, left ventricular assist device.

In LVAD therapy it was previously reported that patients face limited usability of the peripherals, significantly affecting user experience and safety while handling these devices on a daily basis.^11,13–19^ Considering that nearly 70% of LVAD recipients show impaired cognition, 6% have visual dysfunctions, 18% show impaired grip strength and 88% require physical assistance in LVAD battery management,^20^ it is crucial to center the design of LVAD peripherals on the patients who will use them in the outpatient setting, as previously recommended.^11,19,21^ Multiple publications have illustrated the efficacy of iterative simulation-based user studies in refining the design, enhancing user experience, and optimizing the handling properties of medical devices.^22–25^ This iterative approach contributes to the development of next-generation devices that are both more effective and safer in clinical settings.

The study aimed to compare the usability and intuitiveness of LVAD peripherals of the proposed CW peripheral prototypes with clinically available HM3 peripherals in simulated everyday and emergency situations. The primary outcome was the difference in initial success rates and duration to success (DTS) per scenario between the CW and HM3 cohorts. Secondary outcomes included differences in post-scenario survey results, subjective feedback on the device prototypes, and suggestions of potential improvements or identified design issues to enhance a second iteration of the CW peripherals.

## Materials and Methods

This cross-sectional single-center simulation study was conducted in a dedicated simulation center between January 2021 and December 2022, following the methodology previously described.^26^ Six everyday and emergency situations were simulated to evaluate and compare the usability of CW and HM3 LVAD peripherals. The local Institutional Review Board approved the study protocol (identification number: EK2034/2021), the study is in compliance with the ISHLT Ethics Statement, and all participants were able and willing to provide written informed consent.

### Study Population

Former adult LVAD patients (non-HM3 for the HM3 testing cohort) post-heart transplantation (HTX) and non-LVAD-experienced laypeople (LP) were enrolled and tested with either CW or HM3 LVAD peripherals without previous training. This approach accounted for long-term patients who had received initial training but no retraining during LVAD therapy, potentially retaining basic therapy concepts while lacking in-depth emergency management knowledge—as well as LPs, to evaluate the intuitiveness of the devices under testing. Patients who were transplanted more than 10 years before the study, supported by an LVAD for less than 6 months, or with devices other than isolated LVADs were excluded from the study. For the HM3 cohort, subjects previously supported with an HM3 or a HeartMate II pocket controller were also excluded. Subject recruiting aimed to result in balanced numbers of HTX patients and LPs for each tested device (CW *versus* HM3).

### Simulation Setting and Data Collection

**Figure 2.**
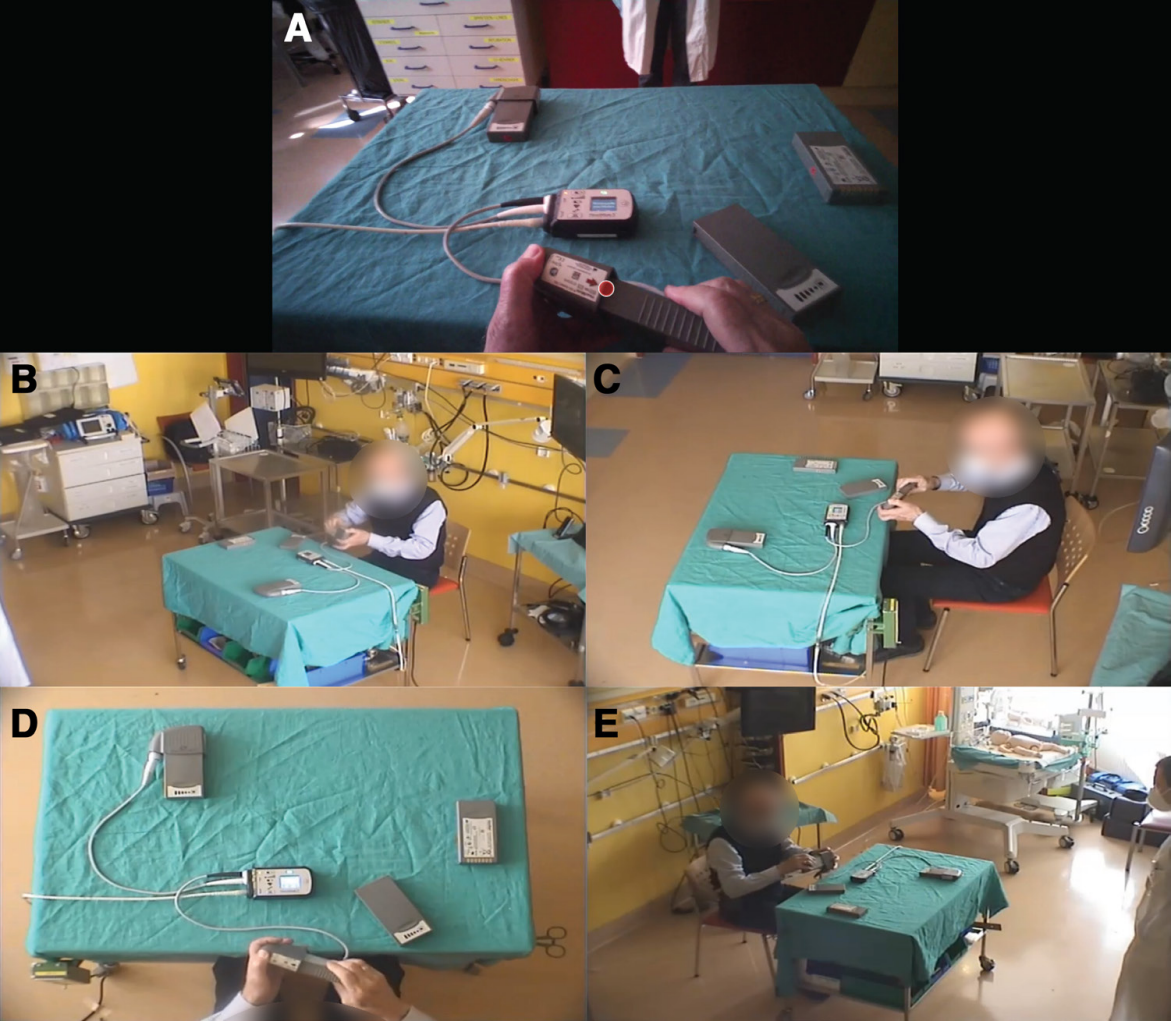
Snapshot of four-perspective room recording (**B–E**) combined with eye tracking recording (**A**) during a HeartMate 3 battery exchange.

The simulation setting was similar to Widhalm *et al*.,^26^ where a four-perspective room recording system (SIMStation Pro, SIMStation GmbH, Vienna, Austria) was used to record the simulated scenarios and subjects were provided with eye tracking glasses (Tobii Pro Glasses 2 and 3; Tobii AB, Stockholm, Sweden), to allow retrospective analysis of gaze behavior. An exemplary snapshot is provided in Figure 2. The comparison of the peripherals’ concepts was based on six defined everyday and emergency situations LVAD patients might encounter^11^: i–iii) battery exchanges in normal or dim light, and within a bag, iv) changing the power supply to alternating current (AC) power, v) disconnecting and reconnecting the driveline, vi) exchanging the controller. All required peripheral components were placed on a table in front of the subjects.

Similar to the previous study,^26^ participants were not given any training before being asked to perform the scenarios. The scenarios were time-capped at 3 min (except for the controller exchange: 5 min), and subjects were granted a second attempt if the first try was unsuccessful. Scenario performance measures included the DTS within two attempts, the number of required attempts, initial success rates, types and number of errors, and pump-off time if applicable. Eye tracking analysis and processing methods are described elsewhere,^26^ with areas of interest defined for relevant and irrelevant LVAD peripheral components for each tested device. Tobii Pro Lab version 1.207 (Tobii AB) was used for post-processing. In case of non-superiority in scenario performance measures for the novel CW peripherals, in-depth eye tracking analysis was conducted for these scenarios including a comparison between initially successful and unsuccessful subjects. Furthermore, subjects completed an 18-item Liker-Scale post-scenario survey described previously^26^ and were asked to comment on the device tested if they desired. Design changes of the CW peripherals were implemented by the manufacturer based on this feedback.

### Statistical Analysis

Statistical analyses were performed with SPSS for Windows Release 29.0.0 (IBM, New York, NY). The assumption of normal distribution was assessed using the Shapiro–Wilk test. Descriptive statistics are presented as mean ± standard deviation (STD) for normally distributed data and as median (interquartile range [IQR]) for non-normally distributed data. Categorical variables are presented as number (percentage). Comparisons between the cohorts (CW *versus* HM3) were performed utilizing unpaired t-test for normally and Mann–Whitney *U* test for non-normally distributed data, Fisher’s exact test for binomial variables, and the Fisher–Freeman–Halton test for variables with more than two categories. The Bonferroni correction was employed to adjust the significance level for multiple comparisons. Statistical significance was set to *p* < 0.05.

## Results

### Subject Characteristics

This study involved 46 subjects (16 CW *vs.* 30 HM3) who completed a total of 276 simulated scenarios. The cohorts comprised former LVAD patients post HTX (52.2%, CW: 56.2% *vs.* HM3: 50.0%) and non-LVAD experienced LP (47.8%, CW: 43.8% *vs.* HM3: 50.0%, *p* = 0.76). Both groups exhibited comparable age distributions (62.5 [11.0] years, *p* = 0.41), gender representation (17.4% female, *p* = 0.69), and body mass index (BMI) (25.4 [5.5] kg/m^2^, *p* = 0.56). Heart transplantation patients had previously been supported for a median duration of 746 (951) days with either the Abbott HeartMate II (CW: 11.1% *vs.* HM3: 13.3%), the Medtronic HVAD (CW: 66.7% *vs.* HM3: 86.7%) or, for the CW cohort, with the HM3 (22.2%), (*p* = 0.17). Detailed demographic information for the full cohort and stratified by tested device is summarized in Table 1.

**Table 1.** Baseline Demographics, Comorbidities, and Highest Completed Education for the Overall Study Population and Stratified by the Two Cohorts: HeartMate 3 *vs.* CorWave LVAD

Variable Median (IQR) or Mean ± STD or n (%)	Full Cohort (n = 46)	CorWave (n = 16)	HeartMate 3* (n = 30)	*p* Value†
Baseline Demographics				
Cohort composition Former LVAD patients post HTX Laypeople	24 (52.2%) 22 (47.8%)	9 (56.2%) 7 (43.8%)	15 (50.0%) 15 (50.0%)	0.76
Age in years	62.5 (11.0)	60.0 (16.0)	63.5 (10.0)	0.41
Gender Male Female	38 (82.6%) 8 (17.4%)	14 (87.5%) 2 (12.5%)	24 (80.0%) 6 (20.0%)	0.69
Height in cm	176.0 (11.0)	179.0 (10.0)	176.0 (10.0)	0.12
Weight in kg	75.5 (21.8)	78.0 (25.1)	75.5 (22.3)	0.97
Body mass index in kg/m^2^	25.4 (5.5)	24.5 (5.5)	25.7 (5.5)	0.56
Dominant arm Right Left Both	42 (91.3%) 2 (4.3%) 2 (4.3%)	14 (87.4%) 1 (6.3%) 1 (6.3%)	28 (93.3%) 1 (3.3%) 1 (3.3%)	1.00
Hand dimensions Hand width in cm Hand length in cm Thumb-to-middle-finger-span in cm Thumb diameter in cm	2.7 ± 0.3 18.5 (2.0) 17.0 ± 1.7 1.8 ± 0.2	2.8 ± 0.3 19.0 (1.9) 16.2 ± 1.7 1.9 ± 0.2	2.6 ± 0.3 18.5 (2.0) 17.3 ± 1.5 1.8 ± 0.2	0.039‡ 0.37‡ 0.12‡ 0.22‡
History of stroke Positive anamnesis Negative anamnesis	3 (6.5%) 43 (93.5%)	2 (12.5%) 14 (87.5%)	1 (3.3%) 29 (96.7%)	0.27
Medical condition affecting scenario completion Arthritis Neuropathy Hearing impairment Mobility impairment Vision impairment	3 (6.5%) 5 (10.9%)s 6 (13.0%) 12 (26.1%) 40 (87.0)	2 (12.5%) 1 (6.3%) 0 (0.0%) 5 (31.3%) 15 (93.8%)	1 (3.3%) 4 (13.3%) 6 (20.0%) 7 (23.3%) 25 (83.3%)	0.54 0.65 0.08 0.73 0.65
Highest completed education Secondary school Vocational school Intermediate vocational school A-levels University degree	3 (6.5%) 10 (21.7%) 9 (19.6%) 12 (26.1%) 12 (26.1%)	0 (0.0%) 3 (18.8%) 5 (31.3%) 5 (31.3%) 3 (18.8%)	3 (10.0%) 7 (23.3%) 4 (13.3%) 7 (23.3%) 9 (30.0%)	0.46
Clinical frailty score CFS 1 CFS 2 CFS 3 CFS 4 CFS 7	2 (4.3%) 27 (58.7%) 11 (23.9%) 5 (10.9%) 1 (2.2%)	1 (6.3%) 6 (37.5%) 6 (37.5%) 3 (18.8%) 0 (0.0%)	1 (3.3%) 21 (70.0%) 5 (16.7%) 2 (6.7%) 1 (3.3%)	0.13
EQ-5D-5L VAS in % Index	85.0 (20.0) 0.99 (0.15)	77.5 (24.0) 0.97 (0.24)	85.0 (16.0) 0.99 (0.10)	0.21 0.47
Demographics Specific to Former LVAD Patients	Full Cohort (n = 24)	CorWave (n = 9)	HeartMate 3 (n = 15)	
NYHA stage NYHA 1 NYHA 2 NYHA 3 NYHA 4	19 (79.2%) 4 (16.7%) 1 (4.2%) 0 (0.0%)	8 (88.9%) 1 (11.1%) 0 (0.0%) 0 (0.0%)	11 (73.3%) 3 (20.0%) 1 (6.7%) 0 (0.0%)	1.00
LVAD type before HTX HeartMate II§ HVAD HeartMate 3	3 (12.5%) 19 (79.2%) 2 (8.3%)	1 (11.1%) 6 (66.7%) 2 (22.2%)	2 (13.3%) 13 (86.7%) 0 (0.0%)	0.17
Days on LVAD support before HTX	746 (951)	831 (1,122)	617 (734)	0.38
Days since HTX	1,889 ± 1,082	1,387 ± 1,084	2,190 ± 996	0.08

* HeartMate 3 cohort has been described elsewhere.^26^

† *p* value comparing CW *vs.* HM3 cohort.

‡ Bonferroni correction for multiple testing.

§ HeartMate II other than Pocket Controller.

CMP, cardiomyopathy; HTX, heart transplantation; IQR, interquartile range; LVAD, left ventricular assist device; NYHA, New York Heart Association; STD, standard deviation; VAS, visual analogue scale.

### Scenario Performance

Study participants conducted 276 scenarios, with 234 successfully completed on the first attempt, indicating significantly higher initial success rates for the CW cohort (92.7% *vs.* 80.6%, *p* = 0.008). Success rates for second attempts were comparable between groups (85.7% *vs.* 83.7%, *p* = 1.00). Subjects required 376.0 (327.0) s to complete all six scenarios within two attempts, whereas participants of the CW group succeeded more than twice as fast as those in the HM3 cohort (219.0 [123.0] s *vs.* 525.5 [289.0] s, *p* < 0.001).

**Figure 3.**
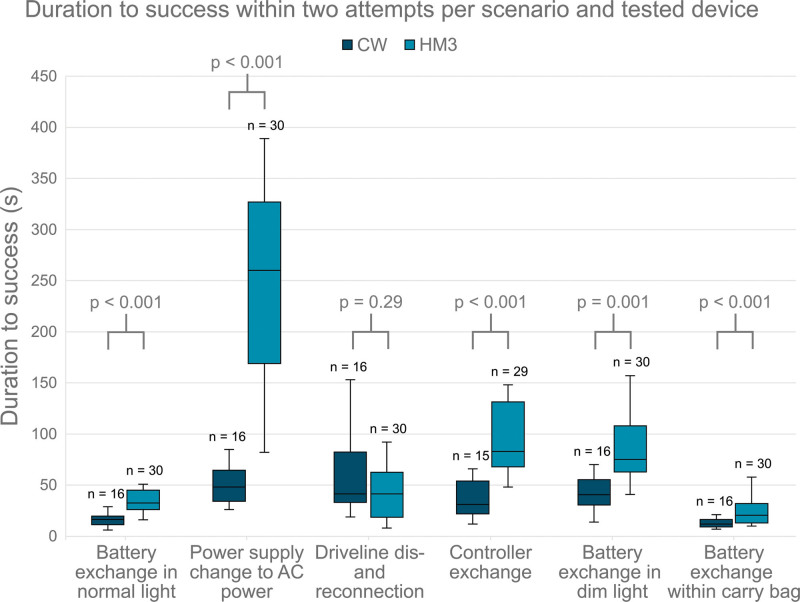
Duration to success within two attempts in seconds per scenario (chronological order) and tested device (HM3: green, CW: blue). CW, CorWave; HM3, HeartMate 3.

Scenario-specific analysis revealed significantly lower DTS for the CW cohort during the battery exchange in normal light (16.5 [8.5] s *vs.* 32.5 [18.8] s, *p* < 0.001) within the carry bag (40.5 [24.8] s *vs.* 75.0 [45.0] s, *p* < 0.001) and in dim light (12.0 [7.3] s *vs.* 21.0 [19.0] s, *p* = 0.001, see Figure 3), even though initial success rates were comparable between both cohorts (*p* ≥ 0.35). The change of power supply from battery to AC power also revealed highest complexity for the HM3 cohort, resulting in a median DTS of 260.0 (158.0) s and 10 subjects (33.0%) unintentionally disconnecting the driveline.^26^ In contrast, the CW peripherals demonstrated greater intuitiveness, with 15 subjects achieving success at first attempt (93.8% *vs.* HM3: 26.7%, *p* < 0.001), showed a five-time lower median DTS of 48.0 (30.3) s (*p* < 0.001), and exhibited significantly fewer handling errors during both attempts (*p* < 0.001). The controller exchange scenario showed comparable initial success rates (*p* = 0.79). However, the CW cohort required less than half the DTS (31.0 [32.0] s *vs.* 83.0 [63.5] s, *p* < 0.001), including significantly reduced pump-off times (12.0 [9.0] s *vs.* 30.0 [49.0] s, *p* = 0.005). When analyzing the driveline dis- and reconnection measures, the CW peripherals were non-inferior to the HM3 in terms of initial success rates (*p* = 0.73), DTS (*p* = 0.29), and pump-off times (*p* = 0.52). A summary of scenario performance measures is provided in Table 2.

**Table 2.** Scenario Performance Measures Stratified by Tested System (CW *vs.* HM3)

Variable Median (IQR) or Mean ± STD or n (%)	CorWave (n = 16)	HeartMate 3* (n = 30)	*p* Value†
Scenario performance			
Battery exchange in normal light			
Success			0.54
First attempt	16 (100.0%)	27 (90.0%)	
Second attempt	0 (0.0%)	3 (10.0%)	
No success	0 (0.0%)	0 (0.0%)	
Duration to success within two attempts	16.5 (8.5) s	32.5 (18.8) s	**<0.001**
Pump disconnection	0 (0.0%)	0 (0.0%)	n.a.
Pump-off time if successful (both attempts)	–	–	n.a.
Any handling errors during first or second attempt	0 (0.0%)	4 (13.3%)	0.28
Power supply change to AC power			
Success			**<0.001**
First attempt Second attempt	15 (93.8%) 1 (6.3%)	8 (26.7%) 17 (56.7%)	
No success	0 (0.0%)	5 (16.7%)	
Duration to success within two attempts	48.0 (30.3) s	260.0 (158.0) s	**<0.001**
Pump disconnection	0 (0.0%)	10 (33.3%)	**0.009**
Pump-off time if successful (both attempts)	–	11.0 (67.3) s	n.a.
Any handling errors during first or second attempt	1 (6.3%)	24 (92.3%)	**<0.001**
Driveline dis- and reconnection			
Success			0.73
First attempt	11 (68.8%)	23 (76.7%)	
Second attempt	5 (31.3%)	7 (23.3%)	
No success	0 (0.0%)	0 (0.0%)	
Duration to success within two attempts	41.5 (49.3) s	41.5 (44.0) s	0.29
Pump disconnection	16 (100.0%)	30 (100.0%)	n.a.
Pump-off time if successful (both attempts)	8.0 (9.0) s	5.0 (7.0) s	0.52
Any handling errors during first or second attempt	6 (37.5%)	10 (33.3%)	0.51
Controller exchange			
Success			0.79
First attempt	15 (93.8%)	27 (90.0%)	
Second attempt	0	2 (6.7%)	
No success	1 (6.3%)	1 (3.3%)	
Duration to success within two attempts	31.0 (32.0) s	83.0 (63.5) s	**<0.001**
Pump disconnection	16 (100.0%)	30 (100.0%)	n.a.
Pump-off time if successful (both attempts)	12.0 (9.0) s	30.0 (49.0) s	**0.005**
Any handling errors during first or second attempt	6 (37.5%)	20 (66.7%)	0.07
Battery exchange within carry bag			
Success			0.35
First attempt	15 (93.8%)	30 (100.0%)	
Second attempt	1 (6.3%)	0.0 (0.0%)	
No success	0.0 (0.0%)	0.0 (0.0%)	
Duration to success within two attempts	40.5 (24.8) s	75.0 (45.0) s	**<0.001**
Pump disconnection	0 (0.0%)	0 (0.0%)	n.a.
Pump-off time if successful (both attempts)	–	–	n.a.
Any handling errors during first or second attempt	1 (6.3%)	2 (6.7%)	1.00
Battery exchange in dim light			
Success			n.a.
First attempt	16 (100.0%)	30 (100.0%)	
Second attempt	0 (0.0%)	0 (0.0%)	
No success	0 (0.0%)	0 (0.0%)	
Duration to success within two attempts	12.0 (7.3) s	21.0 (19.0) s	**0.001**
Pump disconnection	0 (0.0%)	0 (0.0%)	n.a.
Pump-off time if successful (both attempts)	–	–	n.a.
Any handling errors during first or second attempt	0 (0.0%)	5 (16.7%)	0.15

Duration to success provided for participants who were successful within two attempts. Bold values indicates statistically significance at *P* < 0.05.

* HeartMate 3 cohort has been described elsewhere.^26^

† *p* value comparing the cohorts (CW *vs.* HM3).

AC, alternating current; CW, CorWave; HM3, HeartMate 3; IQR, interquartile range; n.a., not applicable; STD, standard deviation.

**Figure 4.**
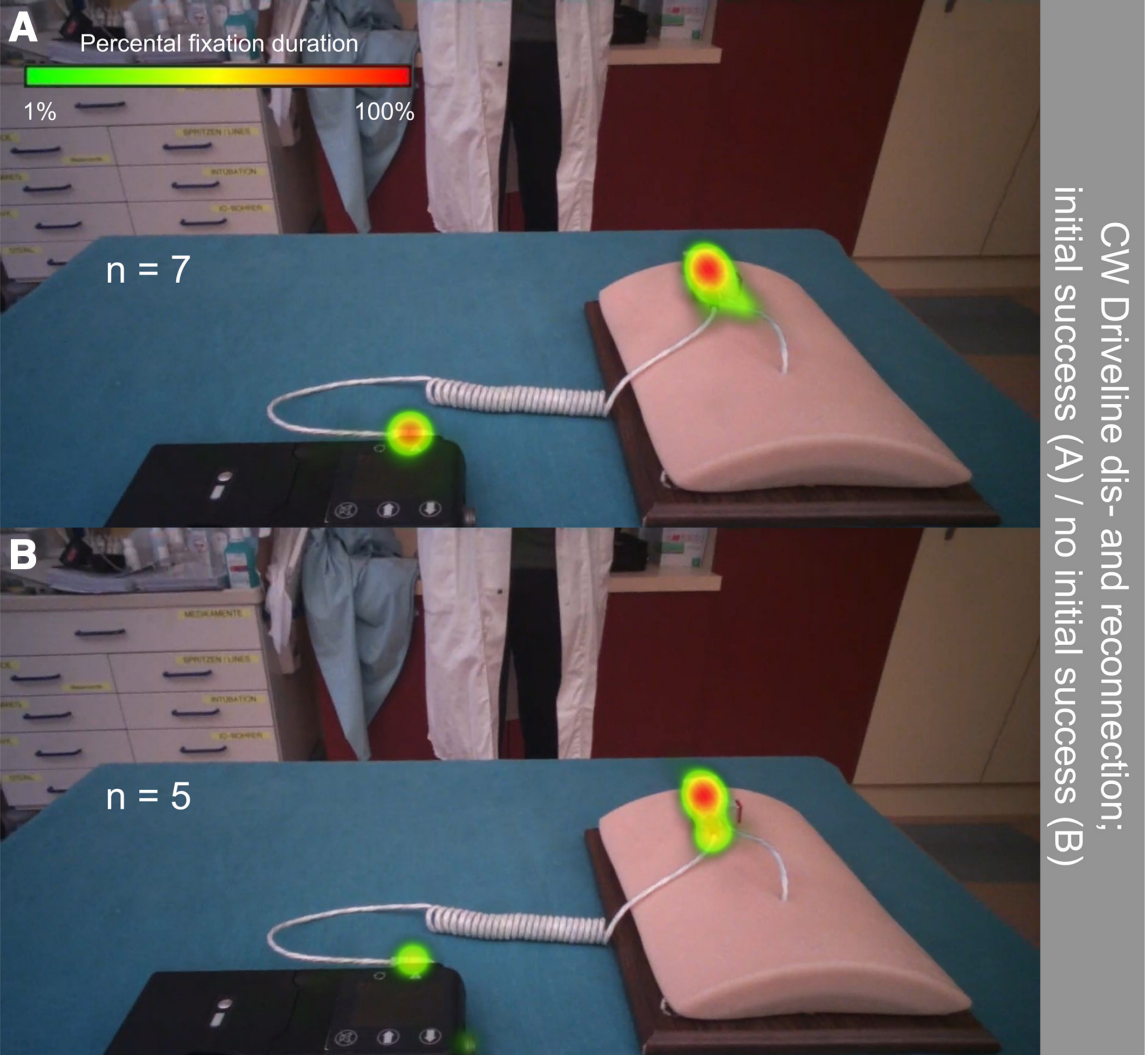
Heat maps for the driveline dis- and reconnection scenario of the CorWave prototype peripherals, generated based on relative fixation duration for initially successful (**A**) and initially unsuccessful (**B**) subjects. CW, CorWave.

Eye tracking analysis comparing initially successful (succ.) and unsuccessful CW subjects of the driveline dis- and reconnection scenario showed no significant differences in percental fixation duration (*p* ≥ 0.17). Both groups mainly focused on the external driveline (not succ: 36.6% [34.4%] *vs.* succ: 24.9 [22.9%], *p* = 0.39) and the driveline connector (not succ: 28.9% [39.8%] *vs.* succ: 38.9% [13.7%], *p* = 0.64) and paid less attention to the controller (not succ. 4.4% [8.5%] *vs.* succ. 0.0% [4.1%], *p* = 0.32), the battery (not succ. 4.4% [8.5%] *vs.* succ. 0.0% [4.1%], *p* = 0.32), the internal driveline (not succ. 1.5% [4.7%] *vs.* succ. 4.8% [19.5%], *p* = 0.29), the abdominal exit-site model (not succ. 0.5% [3.6%] *vs.* succ. 1.4% [1.5%], *p* = 0.59) or other irrelevant components (not succ. 0.3% [6.7%] *vs.* succ. 1.8% [7.7%], *p* = 0.64). The corresponding heat map, displaying the percental fixation duration per group, is provided in Figure 4.

### Survey

**Figure 5.**
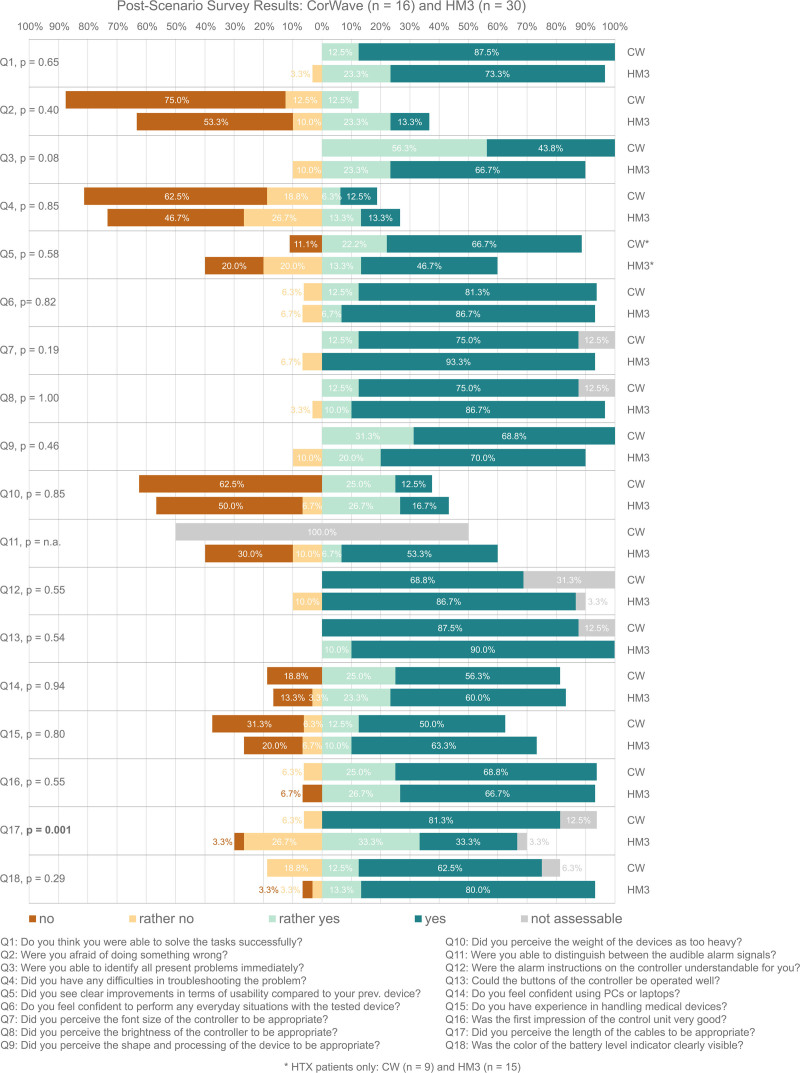
Eighteen-item Likert-Scale post-scenario survey results per device cohort. *p* value comparing CorWave (CW) *vs.* HeartMate 3 (HM3) cohort.

The results of the post-scenario survey are summarized in Figure 5. Significant differences were observed between the CW prototype and HM3 peripherals cohorts regarding cable lengths. In the CW cohort, 81.3% of patients, *vs*. 33.3% of the HM3 cohort, perceived the lengths as appropriate (*p* = 0.001). For all other questions, there was no significant difference between the devices (*p* ≥ 0.08), indicating comparable user experiences. For both systems, a small proportion of participants reported a fear of making a mistake (8.7%) or difficulties with troubleshooting (13%), whereas 84.8% expressed confidence in managing everyday real-world situations. Furthermore, 90.9% and 86.4% of participants fully or rather agreed on the appropriateness of the font size and display brightness, respectively. Although 41.3% of the overall cohort agreed that the peripherals were too heavy, five of the CW-testing HTX patients (55.6%) commented that the peripherals seemed more compact and lightweight than their previously implanted systems, whereas two LPs (28.6%) perceived the components still as too heavy and too big. Overall, 70.9% of HTX participants identified clear usability improvements over their previously implanted LVAD. Seven participants (five HTX, two LP) specifically highlighted the CW helix driveline cable, noting its advantage when dropping the carry bag. Additionally, the CW clip-on battery concept was reported as “very good,” “an improvement” and “easier to handle.” However, three subjects (18.8%) suggested incorporating color coding, asymmetric shapes, or haptic feedback to the batteries to facilitate immediate identification of correct battery orientation during exchanges. Furthermore, two subjects perceived the button controlling the battery charge level LED indicators on the batteries as inappropriate and recommended replacing it with a membrane button, similar to those on the controller. Two subjects also highlighted the benefit of utilizing two battery sizes depending on their activities. Regarding connectors, 10 (62.5%) CW subjects suggested that a customized 90°-bent connector at the controller end of the tethered battery cable would be ideal. Voluntary feedback, including both positive comments and concerns regarding design aspects, was organized by topic and presented in Supplemental Digital Content 2, https://links.lww.com/ASAIO/B523.

### Implications for Design Iteration

**Figure 6.**
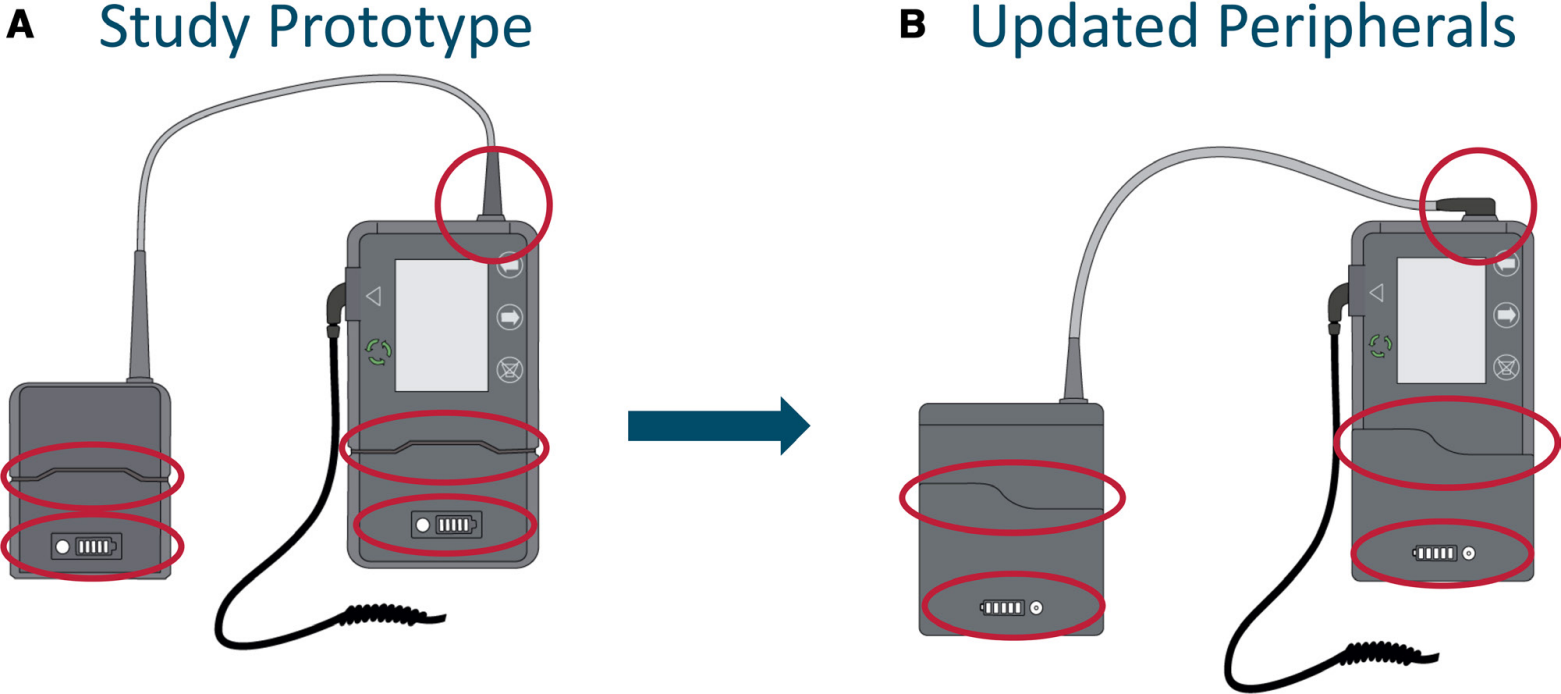
**A**: Prototype of CW peripherals used in the human factor study. **B**: CW peripherals updated after the study. Red circles indicating the main changes: a) 90°-bent connector to minimize space requirements; b) asymmetric shape of the controller-battery connection to guide proper alignment; c) battery level indicator button modified from bare metal to membrane-covered button.

The iterative design process incorporated simulation observations, post-scenario survey feedback, and subjects’ open comments. As a result, the batteries were modified in terms of both shape and battery level indicator (Figure 6): the shape was adjusted from symmetric to asymmetric, allowing easier identification of correct battery orientation, and the battery level indicator button was replaced with a membrane button. Furthermore, the tethered battery connector was altered and rotated by 90° to minimize space requirements. Schematics of the tested CW peripherals prototypes (A) and the next-generation peripherals (B) are provided in Figure 6.

## Discussion

Durable mechanical circulatory support systems demonstrated significant improvements in clinical outcomes during the last decade.^1,27,28^ However, external peripheral components remain burdens in daily activities.^11,13–19^ Furthermore, non-pump-related malfunctions show higher incidence compared to pump failure^29^ and erroneous actions may lead to fatal errors, highlighting the necessity to optimize LVAD peripherals based on a user-centered design approach.^11,19^

This formative human factors study was the first to support an iterative, user-centered LVAD peripherals design approach by comparatively evaluating usability and user experience of a novel concept of LVAD peripherals and a clinically available device^26^ in simulated scenarios. Left ventricular assist device peripherals must be handled and managed by non-healthcare professionals including patients or caregivers, therefore these components should be intuitive and easy to handle, allowing correct operation without previous experience.^14^ This rationale is why this study included 50% LVAD-naive LP and did not incorporate any training phase before the participants were asked to handle the peripherals in the predefined scenarios. Former LVAD patients were included only if they had been supported for at least 6 months before HTX to ensure sufficient handling experience in an outpatient setting. Additionally, former HM3 patients were only included in the CW cohort and patients accustomed to handling the HeartMate II pocket controller were excluded to avoid bias due to similar device concepts.

Even though both cohorts showed initial success rates exceeding 80%, a major finding of this study was that the novel CW peripherals concept appeared to be more intuitive than the clinically available HM3. The CW cohort exhibited 1) higher initial success rates, 2) significantly shorter DTS in all but one scenario, and 3) no unintentional driveline disconnections or fatal controller error due to maloperation.

The possibility to connect the controller additionally to AC power rather than disconnecting both batteries beforehand might be a key aspect for the more intuitive AC power adapter handling of CW peripherals compared to the HM3. This design feature led to significantly higher initial success rates and more than five times lower DTS for the CW cohort, highlighting the largest discrepancies between the two LVAD devices in all scenarios. Battery exchanges were completed twice as quickly with CW peripherals compared to the HM3, however the power concepts differ substantially: CW peripherals use a single clip-on battery with an optional additional tethered battery, comparable to the MVAD PAL controller (Medtronic, Minneapolis, MN) concept.^30^ This design feature may be the next step in offering optional components tailored to individual patient needs.^11,19^

During the AC power scenario, 33% of the HM3 cohort participants unintentionally disconnected the driveline, a mistake that would lead to hazardous situations caused by pump stops for real-life LVAD patients.^26^ In contrast, no disconnections were observed for the CW peripherals. Both the HM3 and the CW peripherals include a connector between the implanted and external parts of the driveline, located a few dozen centimeters from the controller. However, the connection between the external part of the driveline and the controller is fixed in the CW peripherals, whereas HM3 peripherals use a user-actuable connector (Figure 1). All unintentional disconnections happened through this connector. Notably, the power supply in HM3 is replaced by disconnecting the battery or AC power source at the distal end of the cable, rather than near the driveline output on the controller, thereby eliminating the need for intervention at that location. Hence, it could be hypothesized that relocating the driveline connector farther from the controller resulted in a more distinct separation between components essential for daily activities (such as battery exchanges and connection to AC power) and emergency scenarios involving the driveline connection. Moreover, there were no discernable differences in gaze behavior between initially successful and unsuccessful participants in this scenario, suggesting that subjects likely identified the correct components needed to complete the task. Certain scenarios, such as driveline dis- and reconnections and controller exchanges, are typically handled by trained personnel, however caregiving support models vary between centers, and many patients live independently.^31^ Recent evidence from the HM3 system shows that younger patients without social support can manage well on their own,^32^ highlighting the need to assess the usability of these procedures for patients who may face such situations without immediate medical assistance.

The significantly higher satisfaction with the cable length of the CW cohort can be attributed to the shorter and helically coiled driveline, designed to mitigate the impact of accidentally dropping the carry bag by the patient. Besides mechanical characteristics^33^ and appropriate driveline anchoring,^34^ this feature may provide an extra safeguard against driveline infections, which are frequently caused by trauma to the driveline exit site^35^ and may result in serious complications and increased mortality.^36^

Nevertheless, it is important to emphasize that several post-scenario survey results (Figure 5) highlighted the positive aspects of both systems. Only a small proportion of participants reported a fear of making a mistake (8.7%) or experiencing difficulties with troubleshooting (13%), whereas 84.8% felt confident in handling everyday real-world situations. Consistently, no concerns were raised regarding the graphical user interfaces, with 90.9% and 86.4% of participants fully or rather agreeing on the appropriateness of the font sizes and display brightness, respectively. However, this study further contextualized the recent HM3 results^26^ and facilitated a comparison of the currently clinically available peripherals with the novel CW concept. It further emphasizes the essentiality of iterative reevaluation of implemented designs to identify potential redesign necessities.^37^ Despite the excellent clinical results of the HM3,^1,28^ the use of long-term LVAD therapy has not increased in recent years.^1^ Improved usability of LVAD peripherals can significantly increase their application, by several mechanisms: patient comfort, safety, and data integration will make LVADs more appealing to both patients and healthcare providers. These advancements may lead to better clinical outcomes, increased patient satisfaction and greater trust in technology, ultimately leading to more frequent referrals from heart failure cardiologists.

Although this study provided valuable insights into the usability of LVAD peripherals, there are limitations that warrant discussion. First, a preclinical LVAD peripherals prototype was compared to a clinically approved and fully functional device, implicating that the HM3 wearables have already been optimized for clinical use, whereas the CW peripherals were still in preclinical design phase. Therefore, all scenarios focused on participants’ dexterity, whereas other important aspects of LVAD peripherals, such as battery longevity (which directly affects the number of daily exchanges), peripheral durability, and alarm simplicity, were not assessed, as these topics were beyond the scope of this formative study. Second, the formative evaluation was conducted including untrained adult subjects. For further usability testing and the summative evaluation of the final design for clinical use, training and instructions for use should be provided, to show evidence that the CW device is safe and effective to use. Additionally, the novel concept was tested by 16 subjects, leading to unequal sample sizes per cohort. However, previous studies have shown that 15 users can identify at least 90% of usability problems,^38,39^ making this sample size sufficient for formative evaluations. As the only currently approved LVAD on the market, the established HM3 served as a benchmark, with 30 participants included to identify a minimum of 97% and an average of 99% of known use problems.^39^

## Conclusions

This formative human factor evaluation compared the user experience and usability of CW and HM3 LVAD peripherals in simulated every day and emergency situations. The novel CW peripherals concept demonstrated greater intuitiveness, with all but one scenario being solved significantly faster compared to the HM3, which peripherals might benefit from user-centered redesign, considering the complex handling during power supply changes and the hazardous design characteristics of the driveline connector. Involving patients and users early in the design process and conducting formative human factors evaluations can enhance patient-centered design, thereby improving both quality of life and patient safety.

## Acknowledgments

The authors thank all colleagues at the simulation center of the Comprehensive Center for Pediatrics for their support in conducting this study as well as all employees of CorWave SA for providing prototypes of the CorWave LVAD peripherals.

## Supplementary Material
