# Supplementary material for: Enhancing Left Ventricular Assist Device Usability: A Comparative Simulation Study of CorWave and HeartMate 3 Peripherals
Source: ASAIO J. 2025 May 30;72(4):309–19. doi: 10.1097/MAT.0000000000002472 (PMC13021134; doi:10.1097/MAT.0000000000002472)
Supplement: Supplementary file 1 [file mat-72-309-s001.pdf]

Supplementary Material 1: Overview of CorWave controller power and weight specifications, safety and alarm characteristics, and quality of pump data/logfiles

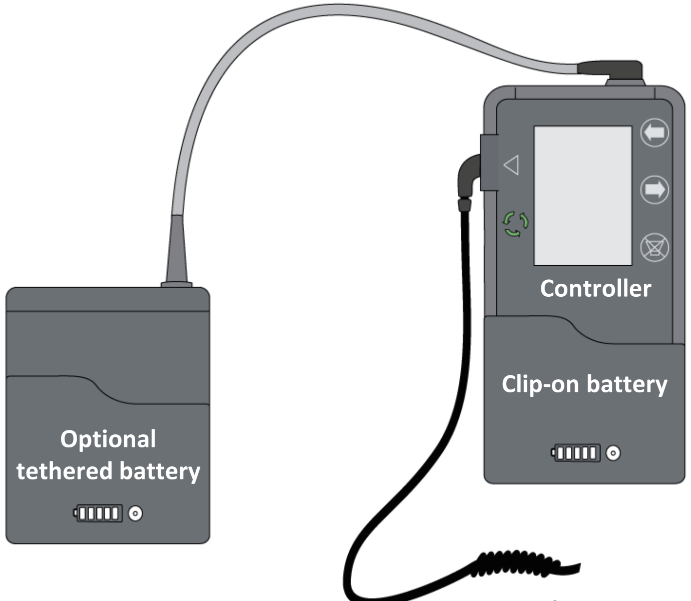

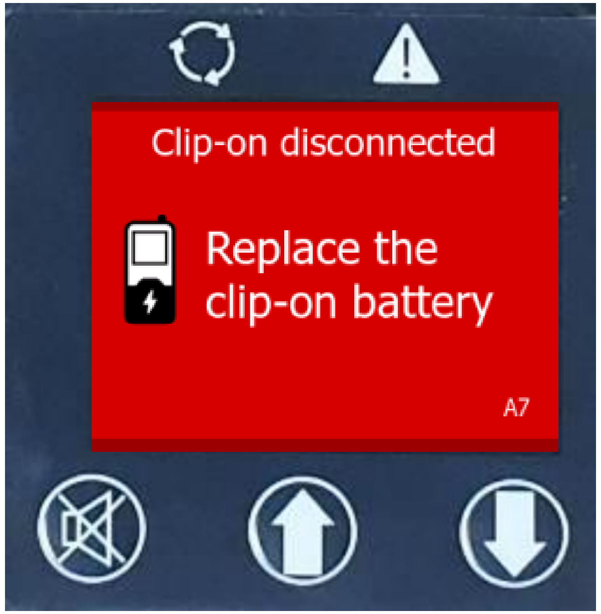

Colored controller screen

SIZE, WEIGHT, AND BATTERY LIFE	
**Controller** (including backup battery)	Length: 13.85 cm, Height: 9 cm, Width: 4 cm<br>Weight: 495 g<br>Backup battery life: 5 min (for clip-on battery exchange)
**External batteries** (clip-on and optional tethered)	Length: 10.2 cm, Height: 9 cm, Width: 5.4 cm<br>Weight: 610 g<br>Batteries life: targeted minimum of 4 hrs in full support at full charge<br>(Combination of clip-on and tethered: 8 hrs)

PERIPHERALS DURABILITY	
**Controller**	The product meets testing standards for use for at least 3 years from the date of first use.
**External batteries** (clip-on and optional tethered)	The product is qualified for 200 charge cycles or a lifespan of 3 years from the date of manufacture, whichever occurs first.

SAFETY AND ALARM CHARACTERISTICS	
**Backup battery** (inside the controller)	Powers the pump for 5 mins during a power-loss emergency or during an intentional clip on battery exchange.
**Alarm activation**	Clip-on battery exchange: Red controller screen requests a battery connection (If the replacement takes > 2 mins, a visual and auditory alert will appear, requesting AC power or battery connection). The alert can be muted, but the sound will reactivate after 2 mins without a power connection.
**Alarm simplicity**	The large, colored screen provides visual alarms (indicating severity: from blue to red), allowing patients to identify and resolve issues independently. For non-solvable issues, a message prompts contacting a clinician, displaying their phone number.

QUALITY OF PUMP DATA/LOGFILES	
**Quality of data**	Utilizing its unique membrane wave technology, the controller records key data on pump performance (mean flow, bpm, power consumption) and algorithm adaptation, including pulsatility levels and suction events.
**Data storage**	Pump support level (equivalent to rpm for rotary pumps) over time following patient activity and clinician settings are stored in the controller to track the self-adaptative algorithm. Sampling interval: 10 sec. Storage capacity: 90 days.
