# Supplementary material for: Enhancing Left Ventricular Assist Device Usability: A Comparative Simulation Study of CorWave and HeartMate 3 Peripherals
Source: ASAIO J. 2025 May 30;72(4):309–19. doi: 10.1097/MAT.0000000000002472 (PMC13021134; doi:10.1097/MAT.0000000000002472)
Supplement: Supplementary file 2 [file mat-72-309-s002.pdf]

# Supplementary Material 2: Voluntary Feedback of Study Participants on LVAD Peripheral Design Aspects

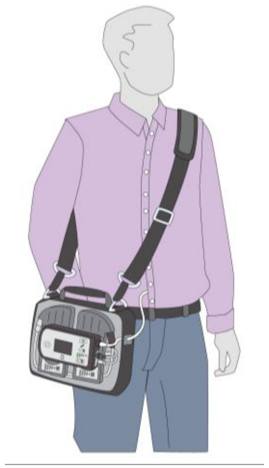

## **HeartMate 3** (HM3)
## (n = 30)

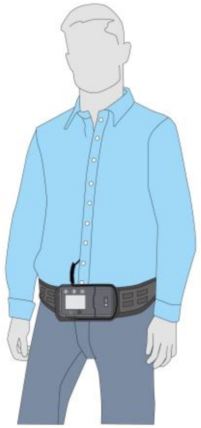

## **CorWave** (CW)
## (n = 16)

### HeartMate 3 (HM3)

#### Pros

1. On battery power, clip-on battery changes are easy
2. The long battery life is convenient
3. The font size is large enough to read alarm message clearly
4. The device is better than my previous LVAD (HVAD)
5. The backup battery in the controller provides safety

#### Cons

1. High complexity to change from battery to AC power
2. Dis- and reconnecting the driveline is very difficult
3. Screw closures are difficult to operate (to connect batteries)
4. Overall, the system is too complex
5. The carry bag could profit from a re-design

### CorWave (CW)

#### Pros

1. The clip-on battery is intuitive to use
2. Helix driveline cable is flexible and safe if controller drops
3. The carrying bag (belt) is comfortable to wear
4. The device is lighter than my previous LVAD
5. The backup battery in the controller provides safety

#### Cons

1. Battery life LED indicators on the batteries inappropriate
2. The tethered battery connector should be 90°-bent
3. Color coding of connectors could be improved
4. The clip-on battery should have asymmetric shapes
5. No haptic feedback implemented (during alarms)
